# Supplementary material for: Temporal nutrition analysis associates dietary regularity and quality with gut microbiome diversity: insights from the Food & You digital cohort
Source: Nat Commun. 2025 Sep 30;16:8635. doi: 10.1038/s41467-025-63799-z (PMC12484809; doi:10.1038/s41467-025-63799-z)
Supplement: Supplementary file 1 — Supplementary Information [file 41467_2025_63799_MOESM1_ESM.pdf]

Temporal nutrition analysis associates dietary regularity and quality with gut microbiome diversity: Insights from the Food & You digital cohort

Rohan Singh[1], *Daniel McDonald*[2], *Alejandra Rios Hernandez*[2], *Se Jin Song*[3], *Andrew Bartko*[2,4], *Rob Knight*[2,3,5,6,7], *Marcel Salathé*[1]

[1] Digital Epidemiology Lab, School of Life Sciences, School of Computer and Communication Sciences, EPFL, Switzerland

[2] Department of Pediatrics, University of California, San Diego, USA

[3] Center for Microbiome Innovation, University of California San Diego, La Jolla, CA, USA

[4] Department of Bioengineering, University of California San Diego, La Jolla, CA, USA

[5] Halıcıoğlu Data Science Institute, University of California San Diego, La Jolla, CA, USA

[6] Shu Chien-Gene Lay Department of Bioengineering, University of California San Diego, La Jolla, CA, USA

[7] Department of Computer Science and Engineering, University of California San Diego, La Jolla, CA, USA

Supplementary:

Metric	Data Type	Description	Input data	Citation
Shannon Entropy	Microbiota	Measures the uncertainty in predicting the species of a randomly selected individual from the dataset	Relative abundance of microbial species in a sample	Shannon et al. 1948
Faith's Phylogenetic Distance	Microbiota	Measures the total branch length of the phylogenetic tree encompassing all members of the community	Phylogenetic tree constructed from 16S rRNA gene sequences	Faith et al. 1992
Observed Features	Microbiota	Counts the number of distinct microbial species present in a sample	Number of unique microbial species detected in a sample	
Pielou's eveness	Microbiota	Measures how evenly the microbial species are distributed in a community	Relative abundance of microbial species in a sample	Pielou et al. 1966
Dietary Diversity Score (HDDS)	Nutrition	Count the number of different food categories consumed	Non-zero food categories consumed in a day	Heidari-Beni et al. 2022
Shannon Entropy	Nutrition	Captures uncertainty in predicting food category of a randomly selected kilocalories	Relative abundance of kilocalories across food categories consumed in a day	Shannon et al 1948, Remans et al. 2014
Simpson index	Nutrition	Probability that two randomly selected kilocalories belong to the same food category	Relative abundance of kilocalories across food categories consumed in a day	Keylock et al. 2005, Borkotoky et al. 2018
Gini-Simpson index	Nutrition	Probability that two randomly selected kilocalories belong to different food categories	Relative abundance of kilocalories across food categories consumed in a day	Borkotoky et al. 2018
Berger-Parker index	Nutrition	Proportion of the most abundant food category relative to the total	Relative abundance of kilocalories across food categories consumed in a day	Khoury et al. 2014
Quantitative Index for Dietary Diversity (QUANTIDD)	Nutrition	Evenness of kilocalorie distribution across food categories	Relative abundance of kilocalories across food categories consumed in a day	Katanoda et al. 2006
Modified Functional Attribute Diversity (MFAD)	Nutrition	Quantifies diversity of nutrients provided by different food items	Carbohydrate, fat, protein, and fiber content (in grams) of eaten each food items	Remans et al., 2014

Supplementary Table 1: Description of various microbiota and dietary diversity metrics.

The specific food categories to generate diet diversity metrics were: '*vegetables*', '*cereals and cereal products*', '*sugary and confectionery*', '*coffee and teas*', '*dairy products*', '*condiments, spices, sauces, and yeast*', '*junk, composite meals and ready-to-eat*', '*fruits*', '*meat products*', '*nuts and seeds*', '*fats and oils*', '*potatoes and other tubers*', '*fruit/vegetable juices*', '*fish and seafood*', '*eggs and egg products*', '*dried legumes*', '*meat and dairy substitutes*', '*other*'.

Multiple Regression Analysis of HEI and Interaction Effects on Gut Microbiome Diversity

We analyzed the relationship between dietary patterns and gut microbiome diversity using multiple linear regression in R (version 4.3.0). The Shannon entropy diversity was used as the dependent variable to measure gut microbiome alpha diversity. Our primary predictor was the HEI, and we examined its interactions with gender and age groups while controlling for relevant covariates. Participants were categorized into three age groups (<35, 35-50, and >50 years). BMI was classified into four categories: underweight, normal (reference), overweight, and obese. Smoking status was categorized as non-smoker (reference), former smoker, and current smoker. Additional covariates included daily eaten quantity (in grams), general hunger level, and daily defecation frequency.

We constructed a multiple linear regression model with three-way interactions between HEI, gender, and age groups. The model was specified as:

$$\textit{Shannon Diversity} \sim \textit{HEI} \times \textit{Gender} \times \textit{Age Group} + \textit{BMI Category} + \textit{Smoking Status} + \textit{Eaten Quantity} + \textit{General Hunger Level} + \textit{Daily Defecation Frequency}$$

Reference categories were set to male gender, age <35 years, normal BMI, and non-smoker status. Visualization of the three-way interaction was performed using the *sjPlot* package in R. Statistical significance was set at $p < 0.05$. The model yielded a residual standard error of 0.501 (958 degrees of freedom) and explained 14.14% of the variance in Shannon diversity (adjusted R-squared = 0.12). The overall model was highly significant ($F(19, 958) = 8.305$, $p < 2.2 \times 10^{-16}$).

Variable	Estimate	Std. Error	t value	P value	Significance
(Intercept)	6.016	0.334	18.013	<2.0e -16	***
HEI	0.011	0.005	2.121	0.034	*
Gender (female)	-0.080	0.445	-0.181	0.857	
Age 35-50	0.231	0.465	0.498	0.619	
Age >50	-0.022	0.623	-0.035	0.972	
BMI (Obese)	-0.189	0.068	-2.787	0.005	**
BMI (Overweight)	-0.100	0.040	-2.525	0.012	*
BMI (Underweight)	-0.101	0.108	-0.940	0.347	
Smoking (former)	-0.121	0.034	-3.522	0.000 4	***
Smoking (current)	-0.179	0.057	-3.157	0.001 6	**
Eaten quantity (g)	-0.00003	0.00002	-1.827	0.068	.
General hunger level	-0.045	0.025	-1.834	0.067	.
Defecation frequency	-0.138	0.023	-6.006	2.7e-09	***
HEI × Gender (female)	0.001	0.007	0.089	0.929	
HEI × Age 35-50	-0.001	0.007	-0.138	0.891	
HEI × Age >50	0.004	0.010	0.389	0.697	

Gender (female) × Age 35-50	-1.013	0.664	-1.527	0.127	
Gender (female) × Age >50	0.348	0.863	0.403	0.687	
HEI × Gender (female) × Age 35-50	0.015	0.010	1.463	0.144	
HEI × Gender (female) × Age >50	-0.006	0.013	-0.461	0.645	

Supplementary Table 2: Multiple linear regression analysis of factors influencing gut microbiome diversity (Shannon entropy), showing main effects and interactions between HEI, gender, and age groups, adjusted for BMI, smoking status, and dietary habits. All hypothesis tests were two-sided. Exact *p* values, 95% confidence intervals and effect sizes are reported for each coefficient.

Multiple Regression Analysis of Diet Quality Regularity on Gut Microbiome Diversity

To further investigate the relationship between daily dietary pattern (Daily HEI) and gut microbiome diversity (Shannon diversity), we performed an additional multiple linear regression analysis (while treating age and BMI as continuous variables) as shown in Supplementary Table 3. We constructed a multiple linear regression model with the following specification:

Shannon Diversity ~ dailyHEI + Gender + BMI + Age + Smoking Status + Eaten Quantity + General Hunger Level + Daily Defecation Frequency

Reference categories were set to male gender and non-smoker status. The model yielded a residual standard error of 0.495 (968 degrees of freedom) and explained 15.5% of the variance in Shannon diversity (adjusted R-squared = 0.147). The overall model was highly significant ($p < 2.2\times10^{-16}$).

Results from this continuous variable model reinforces the importance of diet quality regularity, with daily HEI showing a strong positive association with Shannon diversity ($\beta = 0.019$, $p < 3.58e-13$). For each one-unit increase in daily HEI score, Shannon diversity increased by 0.019 units. Given that HEI ranges

from 0-100, this represents a potential difference of 1.9 units in Shannon diversity across the full spectrum of dietary quality, which is substantial considering the typical range of Shannon diversity in human gut microbiome studies (approximately 2-5).

Variable	Estimate	Std_Error	t_value	P_value	Significance
Intercept	5.691	0.204	28.39	<2.0e-16	***
Daily HEI	0.019	0.003	7.373	3.58e-13	***
Gender (female)	-0.062	0.034	-1.837	0.066	
BMI	-0.009	0.005	-2.07	0.039	*
Age	0.006	0.001	4.611	4.53e-06	***
Smoking (former)	-0.112	0.034	-3.303	0.000991	***
Smoking (current)	-0.156	0.056	-2.805	0.005	**
Eaten quantity (g)	-0.00005	0.00002	-2.536	0.011	*
General hunger level	-0.045	0.024	-1.877	0.06	
Defecation Frequency	-0.134	0.022	-6.044	2.15e-09	***

Supplementary Table 3: Multiple linear regression analysis of factors influencing gut microbiome diversity (Shannon entropy), using continuous variables for BMI and age, and adjusted for smoking status and dietary habits. All hypothesis tests were two-sided. Exact *p* values, 95% confidence intervals and effect sizes are reported for each coefficient.

Validation of CV_{Fruits}-Shannon Diversity Relationship Across Fruit Consumption Ranges

Overall, there is a negative relationship between CV and total fruit consumption, as seen in Supplementary Figure 5A. However, to investigate whether variability in fruit consumption (measured by coefficient of variation, CV) influences gut

microbiome diversity independently of total fruit intake, we conducted a stratified analysis across different ranges of fruit consumption.

We divided the total fruit consumption range (0-400g) into 25g increments, creating 16 narrow ranges to analyze patterns where total fruit consumption was approximately constant. For each range, participants were categorized into CV quintiles (Q1-Q5), and their gut microbiome diversity was classified as either above or below the mean Shannon entropy of the entire cohort. We limited our analysis to ≤400g as consumption values above this threshold had insufficient sample sizes for meaningful comparisons between CV quintiles. This stratification allowed us to examine how CV of fruit consumption relates to microbiome diversity while controlling for total fruit intake. By analyzing the distribution of participants above and below mean Shannon entropy across CV quintiles within each narrow range of fruit consumption, we could assess whether higher CV (Fruits) consistently associated with lower microbiome diversity, independent of total fruit intake.

Supplementary Figure 5C shows that in 75% of the fruit consumption ranges (12 out of 16), the highest CV quintile (Q5) contained more participants below the mean Shannon entropy than above it. This consistent pattern across different levels of total fruit consumption suggests that the variability in fruit intake, rather than just the absolute amount consumed, may be a stronger factor in influencing gut microbiome diversity.

To further validate our findings, we performed a reverse analysis by stratifying participants into ranges of CV (25-200 in 25-unit increments) and examining the relationship between fruit consumption quintiles and Shannon entropy within each CV range, shown in Supplementary Figure 6. This complementary approach allowed us to investigate whether the relationship between fruit consumption and microbiome diversity varied across different levels of consumption regularity. The analysis revealed that in 42.9% (3/7) of CV ranges, participants in the highest fruit consumption quintile (Q5) showed lower microbiome diversity,

a weaker pattern than observed in the primary analysis (75% for high CV). This asymmetry further supports the hypothesis that consumption variability, rather than total intake, may be the stronger driver of microbiome diversity.

Supplementary Figures:

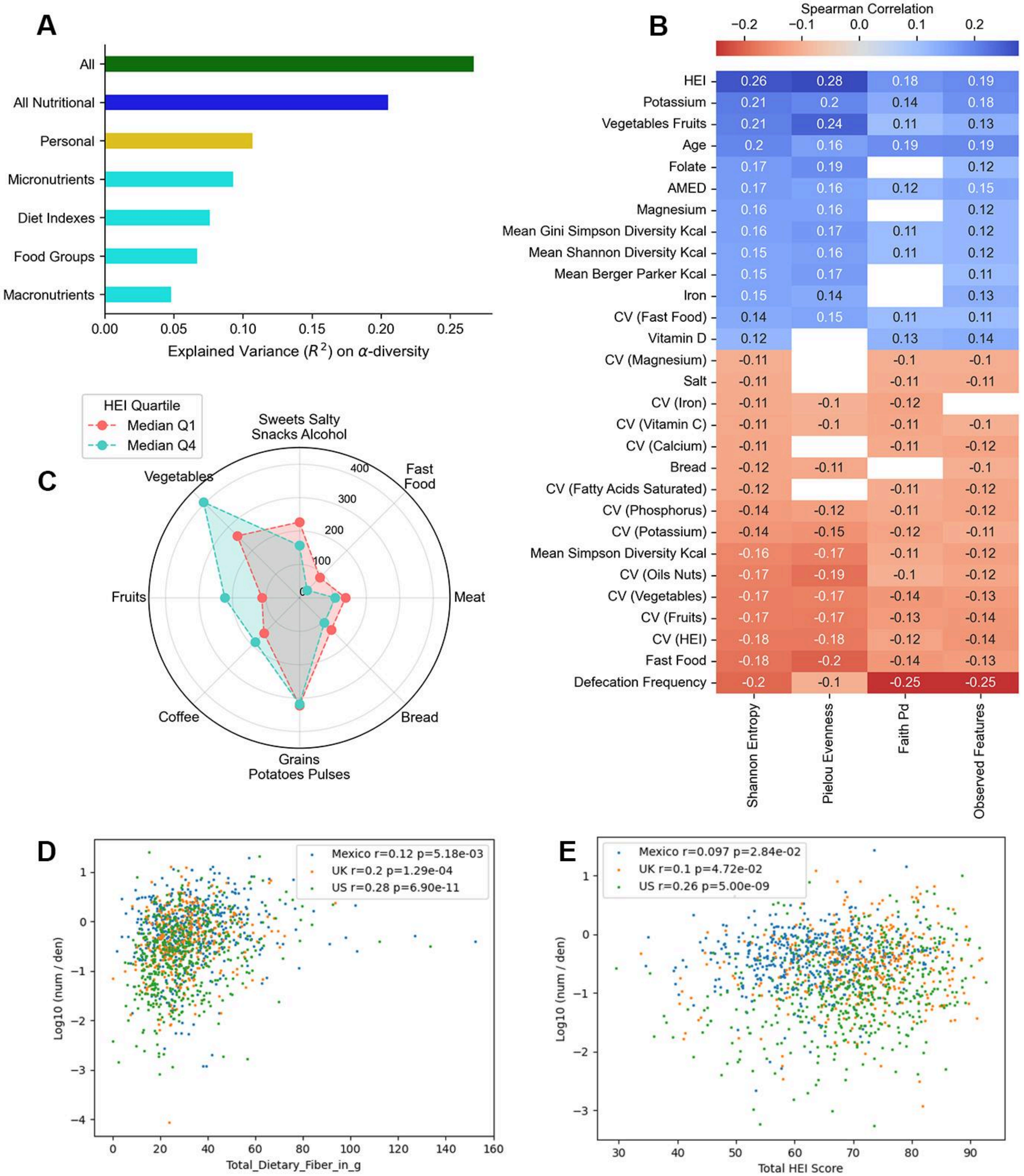

Supplementary Figure 1: Relationships between microbiome diversity, diet, and BMI. (A) The plot shows explained variance (R^2) from linear regression models examining the relationships between Shannon diversity and different variable categories. Categories include combined factors (All), personal characteristics, nutritional components (All Nutritional), and specific dietary variables (micronutrients, diet indexes, food groups, and macronutrients). **(B)** Spearman correlations ($r > 0.1$) of alpha diversities across several nutritional and anthropometric factors. **(C)** Plot highlights the consumption differences (in grams) across various food groups across individuals grouped by extreme quartiles of HEI (i.e., Q1 and Q4). **(D-E)** Correlation of log ratios (of fiber intake and HEI respectively) from the "Food & You" cohort with the US, UK and Mexico "The Human Diet Microbiome Initiative" (THDMI) subset of The Microsetta Initiative .

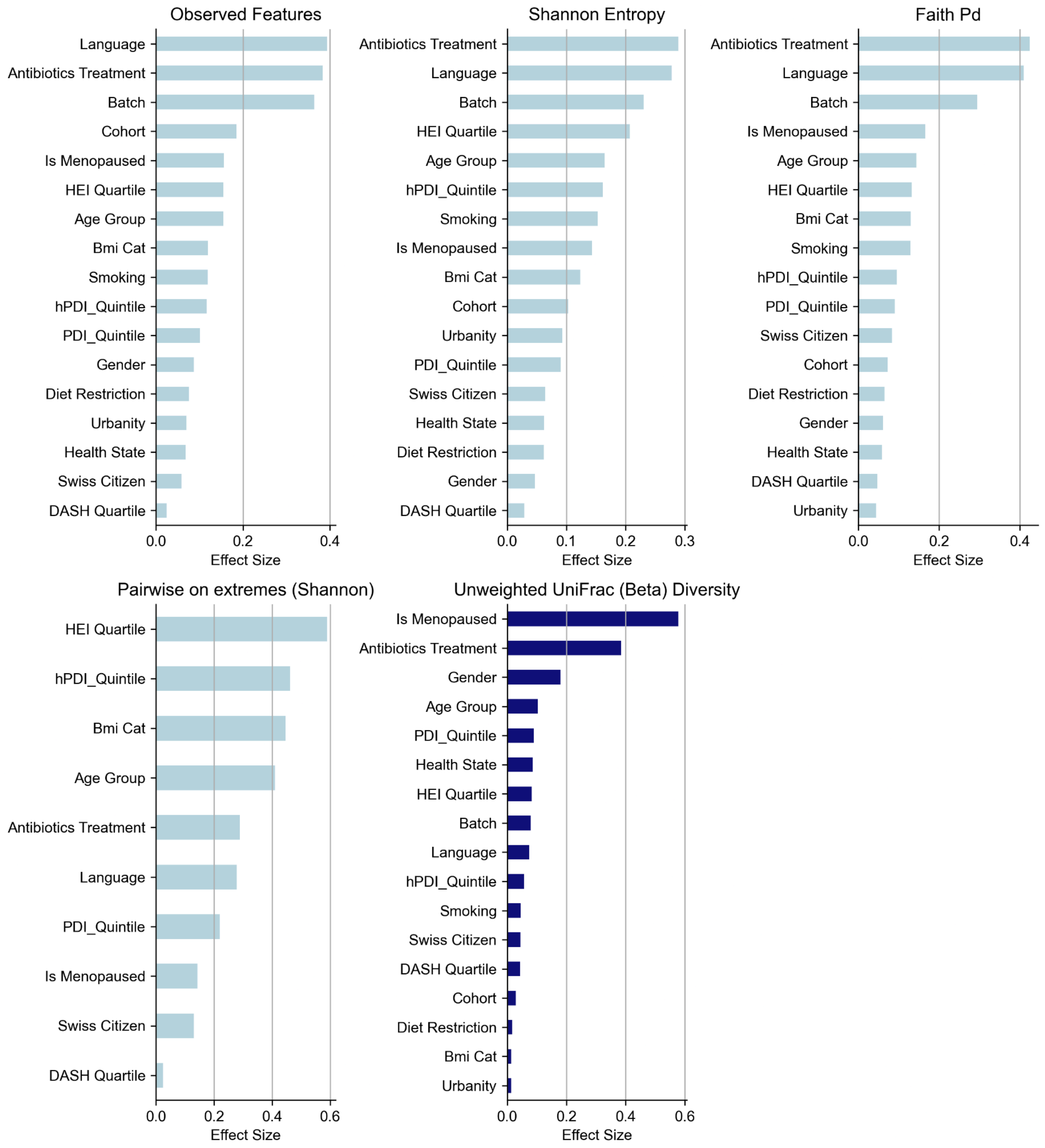

Supplementary Figure 2: Effect sizes of different metadata features on alpha diversity metrics (observed features, Shannon, faith's phylogenetic distance; using Cohen's f for multi-category variables and Cohen's d for binary variables) and beta diversity (unweighted UniFrac; using permutational multivariate effect sizes). Effect sizes were calculated using the *evident* package.

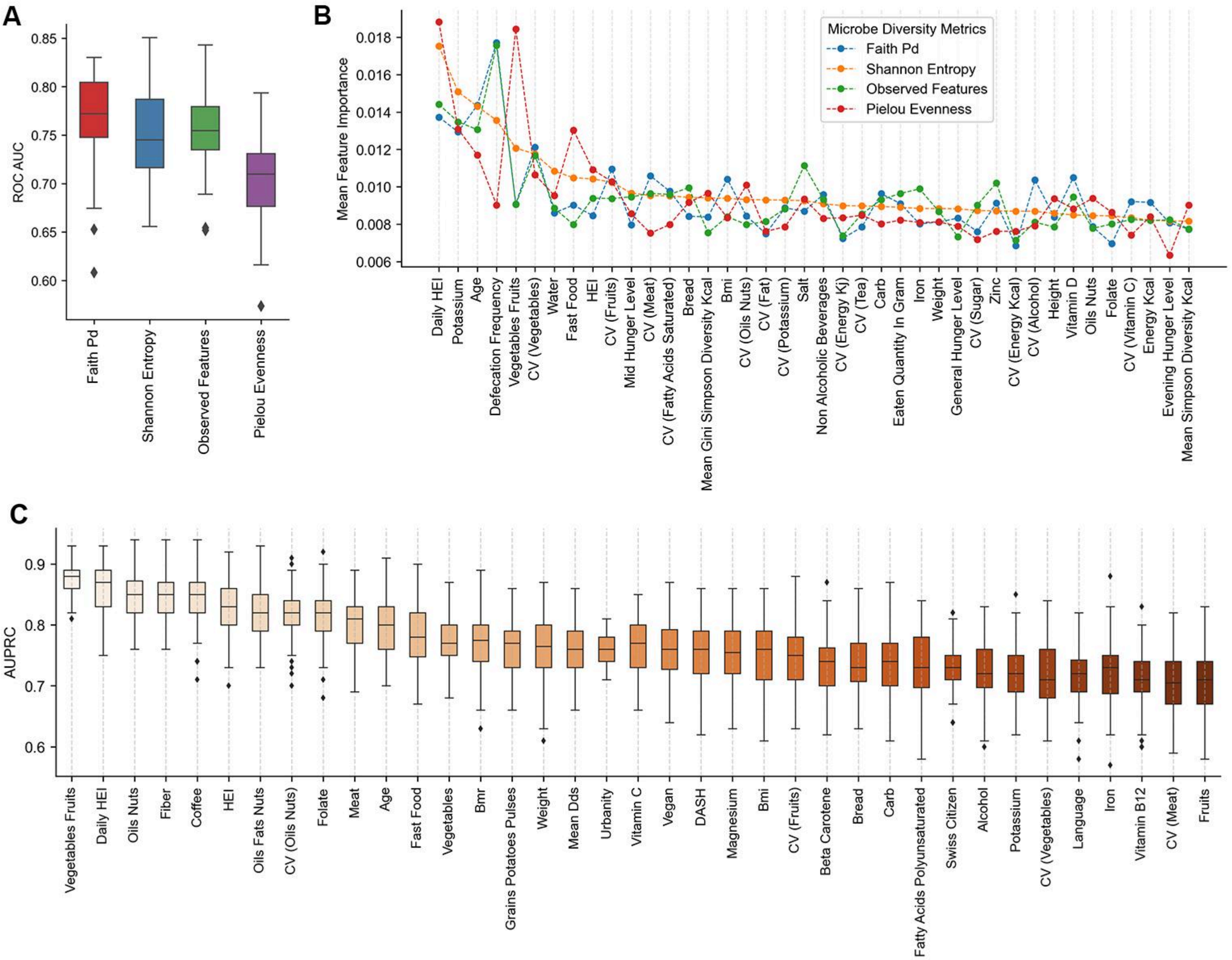

Supplementary Figure 3: Machine learning analysis of alpha diversity predictors and anthropometric and nutritional factors. (A) Performance of classifiers when trained on nutritional and anthropometric features to predict top and bottom quartiles (Q1 vs Q4) of different alpha diversities. Boxplots represent scores across 50 iterations of random training-testing splits (80:20 splits). **(B)** Feature importances of top 40 most prominent factors for the alpha diversity classifiers (sorted by importance on Shannon diversity). (**C**) Area under the precision-recall curve (AUPRC) score for classifiers when trained on microbiota features to predict nutritional and anthropometric features. Each boxplot summarizes 100 independent train-test splits (80:20), with each point corresponding to a unique test set. Boxplots display the median (line), interquartile range (box) and whiskers extending to 1.5× IQR.

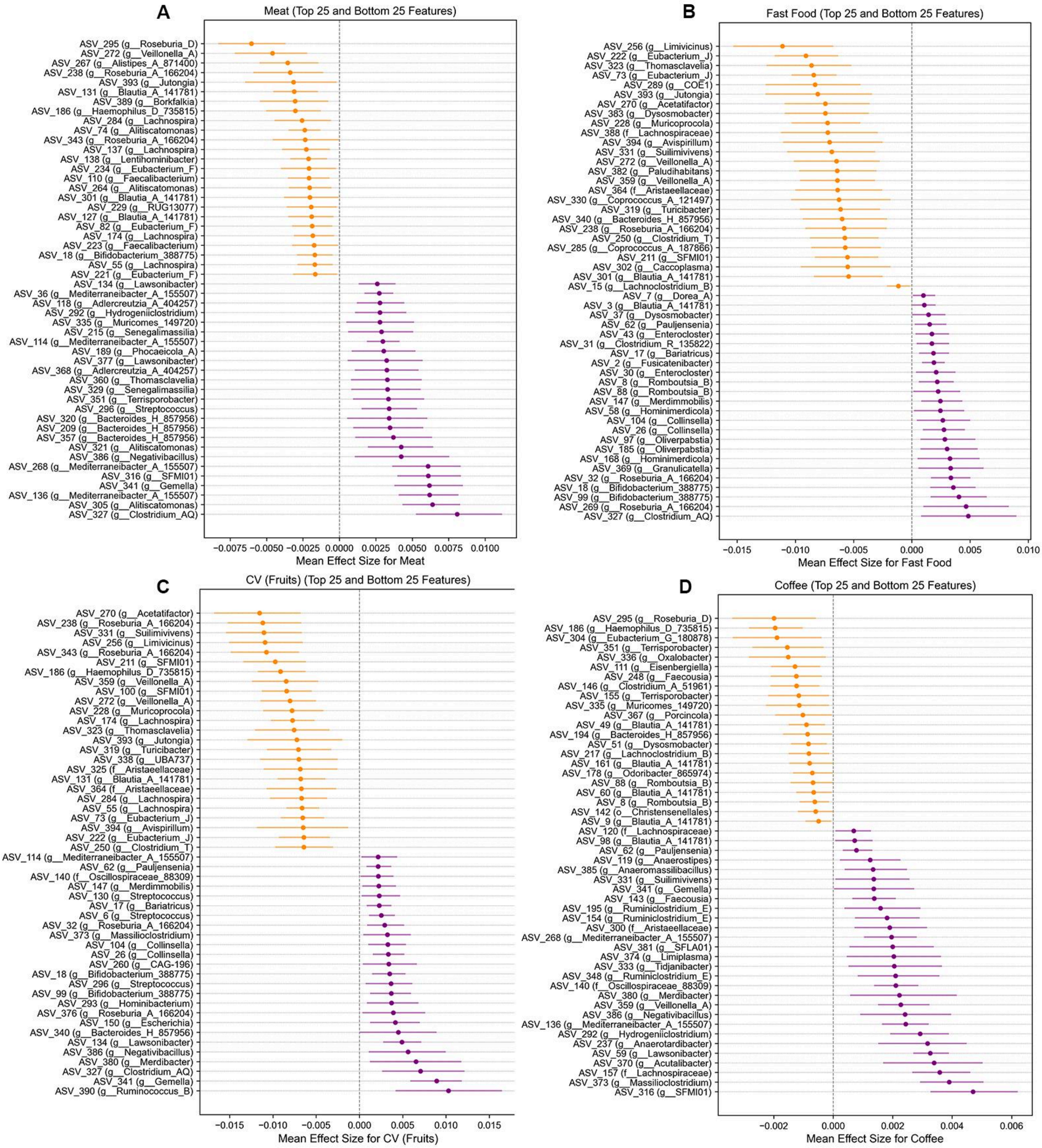
A
Meat (Top 25 and Bottom 25 Features)
ASV_295 (g__Roseburia_D)
ASV_272 (g__Veillonella_A)
ASV_267 (g__Alistipes_A_871400)
ASV_238 (g__Roseburia_A_166204)
ASV_393 (g__Jutongia)
ASV_131 (g__Blautia_A_141781)
ASV_389 (g__Borkfalkia)
ASV_186 (g__Haemophilus_D_735815)
ASV_284 (g__Lachnospira)
ASV_74 (g__Alitiscatomonas)
ASV_343 (g__Roseburia_A_166204)
ASV_137 (g__Lachnospira)
ASV_138 (g__Lentihominibacter)
ASV_234 (g__Eubacterium_F)
ASV_110 (g__Faecalibacterium)
ASV_264 (g__Alitiscatomonas)
ASV_301 (g__Blautia_A_141781)
ASV_229 (g__RUG13077)
ASV_127 (g__Blautia_A_141781)
ASV_82 (g__Eubacterium_F)
ASV_174 (g__Lachnospira)
ASV_223 (g__Faecalibacterium)
ASV_18 (g__Bifidobacterium_388775)
ASV_55 (g__Lachnospira)
ASV_221 (g__Eubacterium_F)
ASV_134 (g__Lawsonibacter)
ASV_36 (g__Mediterraneibacter_A_155507)
ASV_118 (g__Adlercreutzia_A_404257)
ASV_292 (g__Hydrogeniiclostridium)
ASV_335 (g__Muricomes_149720)
ASV_215 (g__Senegalimassilia)
ASV_114 (g__Mediterraneibacter_A_155507)
ASV_189 (g__Phocaeicola_A)
ASV_377 (g__Lawsonibacter)
ASV_368 (g__Adlercreutzia_A_404257)
ASV_360 (g__Thomasclavelia)
ASV_329 (g__Senegalimassilia)
ASV_351 (g__Terrisporobacter)
ASV_296 (g__Streptococcus)
ASV_320 (g__Bacteroides_H_857956)
ASV_209 (g__Bacteroides_H_857956)
ASV_357 (g__Bacteroides_H_857956)
ASV_321 (g__Alitiscatomonas)
ASV_386 (g__Negativibacillus)
ASV_268 (g__Mediterraneibacter_A_155507)
ASV_316 (g__SFMI01)
ASV_341 (g__Gemella)
ASV_136 (g__Mediterraneibacter_A_155507)
ASV_305 (g__Alitiscatomonas)
ASV_327 (g__Clostridium_AQ)
−0.0075 −0.0050 −0.0025 0.0000 0.0025 0.0050 0.0075 0.0100
Mean Effect Size for Meat
B
Fast Food (Top 25 and Bottom 25 Features)
ASV_256 (g__Limivicinus)
ASV_222 (g__Eubacterium_J)
ASV_323 (g__Thomasclavelia)
ASV_73 (g__Eubacterium_J)
ASV_289 (g__COE1)
ASV_393 (g__Jutongia)
ASV_270 (g__Acetatifactor)
ASV_383 (g__Dysosmobacter)
ASV_228 (g__Muricoprocola)
ASV_388 (f__Lachnospiraceae)
ASV_394 (g__Avispirillum)
ASV_331 (g__Suilimivivens)
ASV_272 (g__Veillonella_A)
ASV_382 (g__Paludihabitans)
ASV_359 (g__Veillonella_A)
ASV_364 (f__Aristaeellaceae)
ASV_330 (g__Coprococcus_A_121497)
ASV_319 (g__Turicibacter)
ASV_340 (g__Bacteroides_H_857956)
ASV_238 (g__Roseburia_A_166204)
ASV_250 (g__Clostridium_T)
ASV_285 (g__Coprococcus_A_187866)
ASV_211 (g__SFMI01)
ASV_302 (g__Caccoplasma)
ASV_301 (g__Blautia_A_141781)
ASV_15 (g__Lachnoclostridium_B)
ASV_7 (g__Dorea_A)
ASV_3 (g__Blautia_A_141781)
ASV_37 (g__Dysosmobacter)
ASV_62 (g__Pauljensenia)
ASV_43 (g__Enterocloster)
ASV_31 (g__Clostridium_R_135822)
ASV_17 (g__Bariatricus)
ASV_2 (g__Fusicatenibacter)
ASV_30 (g__Enterocloster)
ASV_8 (g__Romboutsia_B)
ASV_88 (g__Romboutsia_B)
ASV_147 (g__Merdimmobilis)
ASV_58 (g__Hominimerdicola)
ASV_104 (g__Collinsella)
ASV_26 (g__Collinsella)
ASV_97 (g__Oliverpabstia)
ASV_185 (g__Oliverpabstia)
ASV_168 (g__Hominimerdicola)
ASV_369 (g__Granulicatella)
ASV_32 (g__Roseburia_A_166204)
ASV_18 (g__Bifidobacterium_388775)
ASV_99 (g__Bifidobacterium_388775)
ASV_269 (g__Roseburia_A_166204)
ASV_327 (g__Clostridium_AQ)
−0.015 −0.010 −0.005 0.000 0.005 0.010
Mean Effect Size for Fast Food
C
CV (Fruits) (Top 25 and Bottom 25 Features)
ASV_270 (g__Acetatifactor)
ASV_238 (g__Roseburia_A_166204)
ASV_331 (g__Suilimivivens)
ASV_256 (g__Limivicinus)
ASV_343 (g__Roseburia_A_166204)
ASV_211 (g__SFMI01)
ASV_186 (g__Haemophilus_D_735815)
ASV_359 (g__Veillonella_A)
ASV_100 (g__SFMI01)
ASV_272 (g__Veillonella_A)
ASV_228 (g__Muricoprocola)
ASV_174 (g__Lachnospira)
ASV_323 (g__Thomasclavelia)
ASV_393 (g__Jutongia)
ASV_319 (g__Turicibacter)
ASV_338 (g__UBA737)
ASV_325 (f__Aristaeellaceae)
ASV_131 (g__Blautia_A_141781)
ASV_364 (f__Aristaeellaceae)
ASV_284 (g__Lachnospira)
ASV_55 (g__Lachnospira)
ASV_73 (g__Eubacterium_J)
ASV_394 (g__Avispirillum)
ASV_222 (g__Eubacterium_J)
ASV_250 (g__Clostridium_T)
ASV_114 (g__Mediterraneibacter_A_155507)
ASV_62 (g__Pauljensenia)
ASV_140 (f__Oscillospiraceae_88309)
ASV_147 (g__Merdimmobilis)
ASV_130 (g__Streptococcus)
ASV_17 (g__Bariatricus)
ASV_6 (g__Streptococcus)
ASV_32 (g__Roseburia_A_166204)
ASV_373 (g__Massilioclostridium)
ASV_104 (g__Collinsella)
ASV_26 (g__Collinsella)
ASV_260 (g__CAG-196)
ASV_18 (g__Bifidobacterium_388775)
ASV_296 (g__Streptococcus)
ASV_99 (g__Bifidobacterium_388775)
ASV_293 (g__Hominibacterium)
ASV_376 (g__Roseburia_A_166204)
ASV_150 (g__Escherichia)
ASV_340 (g__Bacteroides_H_857956)
ASV_134 (g__Lawsonibacter)
ASV_386 (g__Negativibacillus)
ASV_380 (g__Merdibacter)
ASV_327 (g__Clostridium_AQ)
ASV_341 (g__Gemella)
ASV_390 (g__Ruminococcus_B)
−0.015 −0.010 −0.005 0.000 0.005 0.010 0.015
Mean Effect Size for CV (Fruits)
D
Coffee (Top 25 and Bottom 25 Features)
ASV_295 (g__Roseburia_D)
ASV_186 (g__Haemophilus_D_735815)
ASV_304 (g__Eubacterium_G_180878)
ASV_351 (g__Terrisporobacter)
ASV_336 (g__Oxalobacter)
ASV_111 (g__Eisenbergiella)
ASV_248 (g__Faecousia)
ASV_146 (g__Clostridium_A_51961)
ASV_155 (g__Terrisporobacter)
ASV_335 (g__Muricomes_149720)
ASV_367 (g__Porcincola)
ASV_49 (g__Blautia_A_141781)
ASV_194 (g__Bacteroides_H_857956)
ASV_51 (g__Dysosmobacter)
ASV_217 (g__Lachnoclostridium_B)
ASV_161 (g__Blautia_A_141781)
ASV_178 (g__Odoribacter_865974)
ASV_88 (g__Romboutsia_B)
ASV_60 (g__Blautia_A_141781)
ASV_8 (g__Romboutsia_B)
ASV_142 (o__Christensenellales)
ASV_9 (g__Blautia_A_141781)
ASV_120 (f__Lachnospiraceae)
ASV_98 (g__Blautia_A_141781)
ASV_62 (g__Pauljensenia)
ASV_119 (g__Anaerostipes)
ASV_385 (g__Anaeromassilibacillus)
ASV_331 (g__Suilimivivens)
ASV_341 (g__Gemella)
ASV_143 (g__Faecousia)
ASV_195 (g__Ruminiclostridium_E)
ASV_154 (g__Ruminiclostridium_E)
ASV_300 (f__Aristaeellaceae)
ASV_268 (g__Mediterraneibacter_A_155507)
ASV_381 (g__SFLA01)
ASV_374 (g__Limiplasma)
ASV_333 (g__Tidjanibacter)
ASV_348 (g__Ruminiclostridium_E)
ASV_140 (f__Oscillospiraceae_88309)
ASV_380 (g__Merdibacter)
ASV_359 (g__Veillonella_A)
ASV_386 (g__Negativibacillus)
ASV_136 (g__Mediterraneibacter_A_155507)
ASV_292 (g__Hydrogeniiclostridium)
ASV_237 (g__Anaerotardibacter)
ASV_59 (g__Lawsonibacter)
ASV_370 (g__Acutalibacter)
ASV_157 (f__Lachnospiraceae)
ASV_373 (g__Massilioclostridium)
ASV_316 (g__SFMI01)
−0.002 0.000 0.002 0.004 0.006
Mean Effect Size for Coffee

Supplementary Figure 4: Differential abundance analysis of ASVs associated with four prominent dietary factors. (A-D) The top 25 positively (purple) and bottom 25 negatively (orange) associated ASVs for meat (A), fast food (B), Coefficient of Variation for Fruits (C), and coffee (D) consumption patterns. Points indicate mean effect sizes, and error bars represent the highest density interval (HDI) for each taxon's association with the respective dietary component. Only ASVs with credible intervals not crossing zero are shown. The full set of credible taxa can be found in Supplementary Data 1.

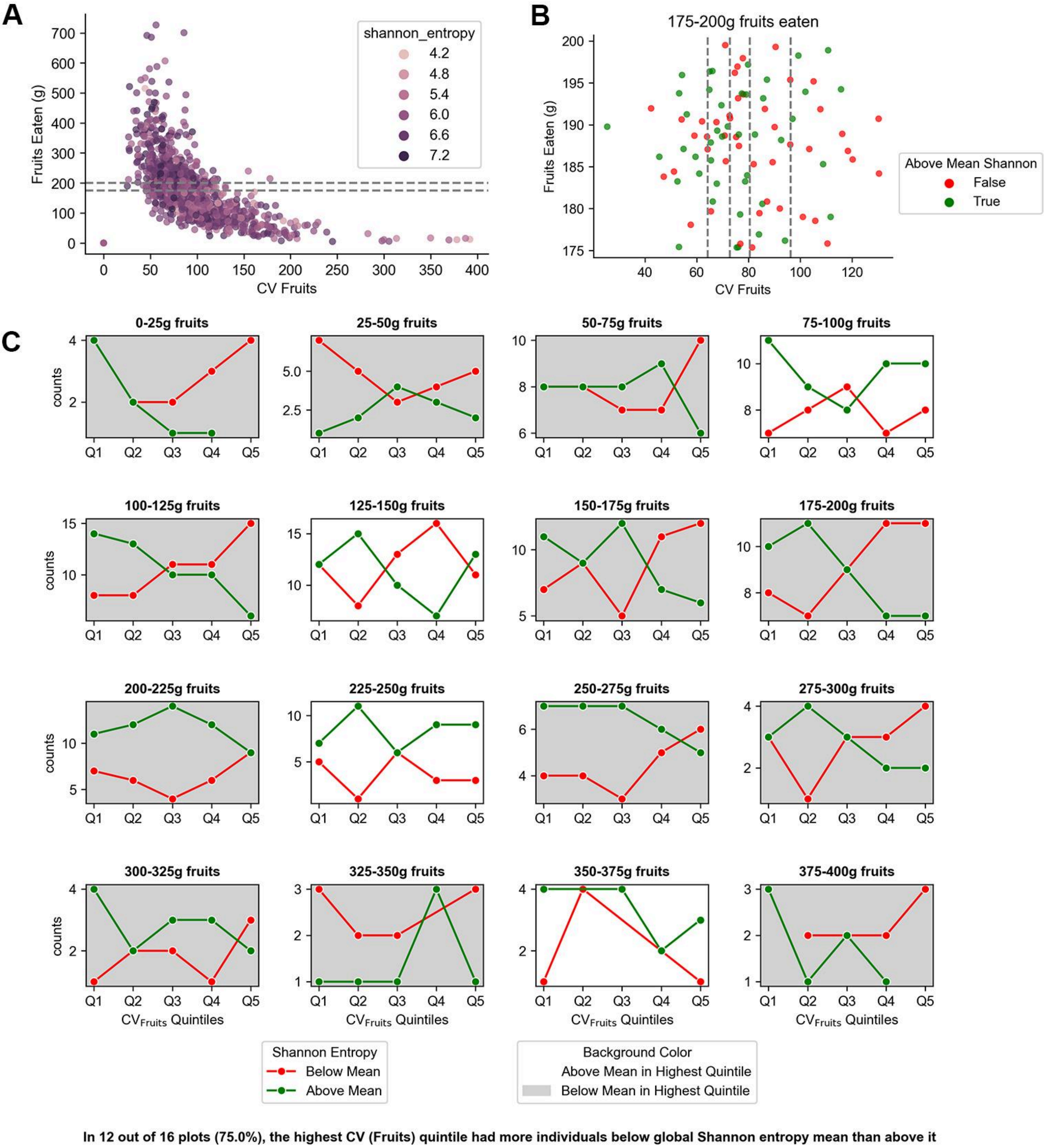

Supplementary Figure 5: (A) Relationship between coefficient of variation (CV) of fruit intake and total fruits eaten (g), colored by microbiota Shannon entropy. Dotted lines reflect an example chunk for 175-200g of fruits eaten. **(B)** Detailed view of participants consuming 175-200g of fruits,

showing CV distribution stratified by above (green) or below (red) mean Shannon entropy. Vertical dashed lines represent CV quintiles. **(C)** Distribution of participants with above (green) and below (red) mean Shannon entropy across CV quintiles (Q1-Q5) for different ranges of fruit consumption (25g increments from 0-400g). Grey background indicates ranges where the highest CV quintile (Q5) shows more participants below mean Shannon entropy than above it, occurring in 75% (12/16) of the ranges and suggesting a consistent association between higher fruit intake variability and lower microbial diversity.

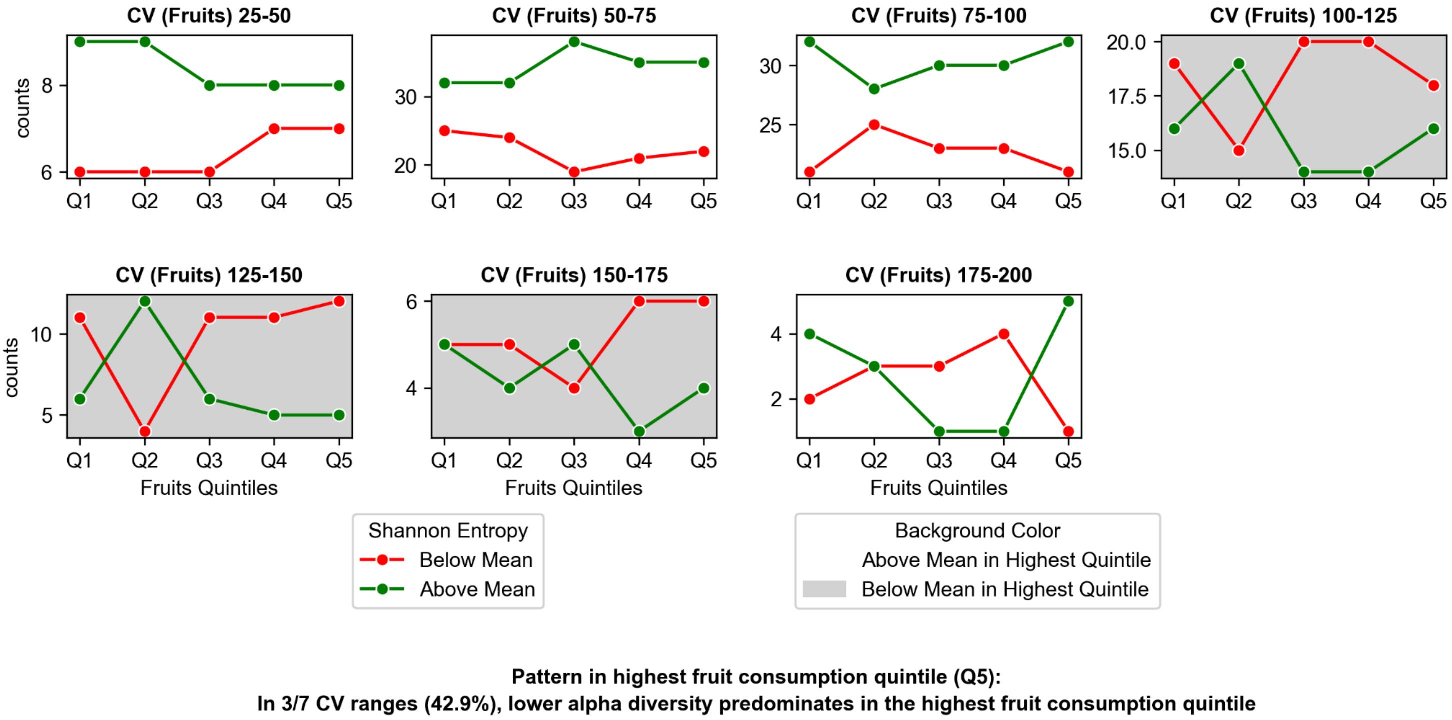

Supplementary Figure 6: Analysis of fruit consumption effects on microbiome diversity within constant CV ranges. Each panel represents a 25-unit range of coefficient of variation (CV) for fruit consumption, showing the distribution of participants with above (green) and below (red) mean Shannon entropy across fruit consumption quintiles (Q1-Q5). Grey backgrounds indicate ranges where the highest fruit consumption quintile (Q5) shows more participants below mean Shannon entropy than above it, occurring in 42.9% (3/7) of the CV ranges.

Supplementary References:

Shannon, Claude Elwood. "A mathematical theory of communication." *ACM SIGMOBILE mobile computing and communications review* 5.1 (2001): 3-55.

Faith, Daniel P. "Conservation evaluation and phylogenetic diversity." *Biological conservation* 61.1 (1992): 1-10.

DeSantis, Todd Z., et al. "Greengenes, a chimera-checked 16S rRNA gene database and workbench compatible with ARB." *Applied and environmental microbiology* 72.7 (2006): 5069-5072.

Pielou, Evelyn C. "The measurement of diversity in different types of biological collections." *Journal of theoretical biology* 13 (1966): 131-144.

Heidari-Beni, Motahar, Zeinab Hemati, and Mostafa Qorbani. "The dietary diversity score: methods, indicators, and applications to general population." *Biomarkers in Nutrition*. Cham: Springer International Publishing, 2022. 293-306.

Remans, Roseline, et al. "Measuring nutritional diversity of national food supplies." *Global Food Security* 3.3-4 (2014): 174-182.

Keylock, CJ□. "Simpson diversity and the Shannon–Wiener index as special cases of a generalized entropy." *Oikos* 109.1 (2005): 203-207.

Borkotoky, Kakoli, Sayeed Unisa, and Ashish Kumar Gupta. "State-level dietary diversity as a contextual determinant of nutritional status of children in India: a multilevel approach." *Journal of biosocial science* 50.1 (2018): 26-52.

Khoury, Colin K., et al. "Increasing homogeneity in global food supplies and the implications for food security." *Proceedings of the national Academy of Sciences* 111.11 (2014): 4001-4006.

Katanoda, Kota, Hee-Seon Kim, and Yasuhiro Matsumura. "New Quantitative Index for Dietary Diversity (QUANTIDD) and its annual changes in the Japanese." *Nutrition* 22.3 (2006): 283-287.
