# Supplementary material for: Temporal nutrition analysis associates dietary regularity and quality with gut microbiome diversity: insights from the Food & You digital cohort
Source: Nat Commun. 2025 Sep 30;16:8635. doi: 10.1038/s41467-025-63799-z (PMC12484809; doi:10.1038/s41467-025-63799-z)
Supplement: Supplementary file 2 — Description of Additional Supplementary Files [file 41467_2025_63799_MOESM2_ESM.pdf]

File Name: Supplementary Data 1
Description: Results of BIRDMAn differential abundance analysis. Table includes taxon identifiers, effect size estimates, highest density intervals (HDI), standard deviations, credible status, associated dietary variables, and representative sequences for amplicon sequence variants (ASVs).

File Name: Supplementary Data 2
Description: XGBoost model results showing classifier (AUROC, AUPRC), regressor (Spearman), and feature importance outputs across 100 iterations for dietary and lifestyle variables.
