# Supplementary material for: Temporal nutrition analysis associates dietary regularity and quality with gut microbiome diversity: insights from the Food & You digital cohort
Source: Nat Commun. 2025 Sep 30;16:8635. doi: 10.1038/s41467-025-63799-z (PMC12484809; doi:10.1038/s41467-025-63799-z)
Supplement: Supplementary file 5 — Reporting Summary [file 41467_2025_63799_MOESM5_ESM.pdf]

nature portfolio

Corresponding author(s): Dr. Marcel Salathé

Last updated by author(s): Aug 15, 2025

Reporting Summary

Nature Portfolio wishes to improve the reproducibility of the work that we publish. This form provides structure for consistency and transparency in reporting. For further information on Nature Portfolio policies, see our Editorial Policies and the Editorial Policy Checklist.

Statistics

For all statistical analyses, confirm that the following items are present in the figure legend, table legend, main text, or Methods section.

n/a	Confirmed	
☐	☒	The exact sample size (*n*) for each experimental group/condition, given as a discrete number and unit of measurement
☐	☒	A statement on whether measurements were taken from distinct samples or whether the same sample was measured repeatedly
☐	☒	The statistical test(s) used AND whether they are one- or two-sided<br>*Only common tests should be described solely by name; describe more complex techniques in the Methods section.*
☐	☒	A description of all covariates tested
☐	☒	A description of any assumptions or corrections, such as tests of normality and adjustment for multiple comparisons
☐	☒	A full description of the statistical parameters including central tendency (e.g. means) or other basic estimates (e.g. regression coefficient) AND variation (e.g. standard deviation) or associated estimates of uncertainty (e.g. confidence intervals)
☐	☒	For null hypothesis testing, the test statistic (e.g. *F*, *t*, *r*) with confidence intervals, effect sizes, degrees of freedom and *P* value noted<br>*Give P values as exact values whenever suitable.*
☐	☒	For Bayesian analysis, information on the choice of priors and Markov chain Monte Carlo settings
☐	☒	For hierarchical and complex designs, identification of the appropriate level for tests and full reporting of outcomes
☐	☒	Estimates of effect sizes (e.g. Cohen's *d*, Pearson's *r*), indicating how they were calculated

Our web collection on statistics for biologists contains articles on many of the points above.

Software and code

Policy information about availability of computer code

Data collection	MyFoodRepo mobile app - developed by Digital Epidemiology Lab for food logging and dietary intake data collection.<br>Food data collection validation: Expert human annotation system for validating all food entries in MyFoodRepo<br><br>Microbiome sequencing:<br>-Performed at Microsynth AG (Switzerland)<br>-Used V4 region of 16S rRNA gene amplification with 515F and 806R primers
Data analysis	QIIME 2 (version 2024.2) for microbiome preprocessing<br>Deblur (version 2024.2) for denoising and constructing amplicon sequence variants<br>Greengenes2 database (version 2024.09) for taxonomic classification<br>BIRDMAn for differential abundance analysis<br>R (version 4.3.0)<br>python (version 3.8.18)<br>XGBoost (version 2.0.3)<br><br>The analysis code used to generate the results is publicly available on GitHub (https://github.com/digitalepidemiologylab/dietary-consistency-and-quality-associated-with-gut-microbiota-diversity-paper), and archived at Zenodo (DOI: 10.5281/zenodo.16779103).

For manuscripts utilizing custom algorithms or software that are central to the research but not yet described in published literature, software must be made available to editors and reviewers. We strongly encourage code deposition in a community repository (e.g. GitHub). See the Nature Portfolio guidelines for submitting code & software for further information.

Data

Policy information about availability of data

All manuscripts must include a data availability statement. This statement should provide the following information, where applicable:

- Accession codes, unique identifiers, or web links for publicly available datasets
- A description of any restrictions on data availability
- For clinical datasets or third party data, please ensure that the statement adheres to our policy

16S rRNA gene sequencing data from this study are publicly available in the European Nucleotide Archive (ENA) under accession number PRJEB85942 and in Qiita under study ID 15880. Metadata containing clinical, demographic, and nutritional variables cannot be deposited publicly due to participant privacy and ethical restrictions. Access to this metadata can be requested by contacting the corresponding author, subject to institutional ethical compliance.

Research involving human participants, their data, or biological material

Policy information about studies with human participants or human data. See also policy information about sex, gender (identity/presentation), and sexual orientation and race, ethnicity and racism.

Reporting on sex and gender	Sex (biological attribute) was determined through self-reporting during enrollment questionnaire. The study included:<br><br>Cohort B: Equal distribution of biological males and females<br>Cohort C: Restricted to biological females of reproductive age who did not use hormonal contraceptives<br><br>Sex-specific analyses were performed, with sex included as a biological variable in multiple regression analyses examining associations between diet quality, microbiota diversity, and health outcomes (see Supplementary Tables 2 and 3). All relevant data were disaggregated by sex, with consent obtained for sharing individual-level data.<br>Gender (social and cultural) data were not specifically collected or analyzed in this study, as the focus was on biological factors affecting gut microbiota and dietary patterns. Where we refer to 'gender' in the manuscript, this reflects the biological sex of participants.
Reporting on race, ethnicity, or other socially relevant groupings	Participants were categorized by citizenship status (Swiss, Binational, or Foreigner) and linguistic region (German or Latin - including French, Italian, and Romansh-speaking regions) based on self-reporting in questionnaires. These categories were used to examine potential cultural differences in dietary patterns across Switzerland's diverse regions, not as proxies for other variables.
Population characteristics	Food and You is a digital nutrition cohort comprising of participants across Switzerland from 2018-2022. The study focused on healthy participants who tracked their dietary intake for 2-4 weeks. Key characteristics included:<br><br>-Age groups (18-35, 35-50, >50 years)<br>-BMI categories (underweight, normal, overweight, obese)<br>-Smoking status (non-smoker, former smoker, current smoker)<br>-Gender (male/female)<br>-Linguistic region (German/Latin)<br>-Citizenship status (Swiss/Binational/Foreigner)<br>-Smoking status (non-smokers, former and current smokers)<br>-Past antibiotic usage<br>-Menopause status was recorded for relevant participants
Recruitment	Participants were recruited as part of the "Food & You" digital cohort study from 2018 to 2022 in Switzerland. The study was advertised through social media channels, leading to potential self-selection bias toward younger, digitally-savvy participants with scientific interests. The requirement to track food intake, use continuous glucose monitors, and provide stool samples may have attracted participants particularly interested in health and nutrition, potentially introducing self-selection bias toward health-conscious individuals. Indeed, the study population shows higher education levels and healthier lifestyle indicators compared to the general Swiss population.
Ethics oversight	The study protocol was approved by the Geneva Ethics Commission (approval number: 2017-02124) and registered with both the Swiss Federal Office of Public Health (SNCTP000002833) and ClinicalTrials.gov (NCT03848299).

Note that full information on the approval of the study protocol must also be provided in the manuscript.

Field-specific reporting

Please select the one below that is the best fit for your research. If you are not sure, read the appropriate sections before making your selection.

[x] Life sciences [ ] Behavioural & social sciences [ ] Ecological, evolutionary & environmental sciences

For a reference copy of the document with all sections, see nature.com/documents/nr-reporting-summary-flat.pdf

Life sciences study design

All studies must disclose on these points even when the disclosure is negative.

Sample size	The study included 1,014 participants from Switzerland. No formal sample size calculation was performed as this was an observational study. After quality control steps (excluding participants with <5 tracking days and those with daily energy intake below 1,000 kcal), 978 participants were retained for dietary analyses. For microbiome analyses, samples were rarefied to 15k reads, retaining 992 samples.The sample size is comparable to other large-scale diet-microbiome studies (e.g., Asnicar et al. 2021, n>1,000) and provided sufficient statistical power to detect associations between dietary patterns and microbiota diversity.
Data exclusions	Exclusion Criteria:<br><br>Pregnancy.<br>Persons on dialysis.<br>Persons with chronic immunosuppressive medication usage.<br>persons with skin disease, including contact dermatitis<br>Critically-ill patients.<br>Breastfeeding.<br>Usage of antibiotics in the three months prior to enrollment.<br>Chronically active inflammatory or neoplastic disease in the three years prior to enrollment.<br>Chronic gastrointestinal disorder, including Inflammatory Bowel Disease and Celiac disease.<br>Active neuropsychiatric disorder.<br>Myocardial infarction or cerebrovascular accident in the six months prior to enrollment.<br>Pre-diagnosed type I or type II diabetes mellitus.<br><br>For analysis of this paper:<br>-Tracking days with total energy intake below 1,000 kcal<br>-Participants with fewer than 5 remaining tracking days<br>-For microbiome analyses, samples with insufficient sequencing depth (<15k reads)<br>-For stool quality analyses, participants reporting fewer than 5 days of stool quality data
Replication	As this was an observational cohort study examining associations between diet and microbiota in a single population, direct experimental replication was not applicable. However, we validated our findings by:<br><br>-Comparing our results with other geographical cohorts (US, UK, and Mexico)<br>-Using multiple independent statistical approaches (correlation analysis, differential abundance analysis, machine learning)
Randomization	This was an observational study without experimental groups, so randomization was not applicable. Potential confounding variables (age, BMI, gender, smoking status) were controlled for in statistical analyses.
Blinding	This was an observational study without intervention groups, so blinding was not applicable. However, microbiome sequencing and bioinformatic analyses were performed independently from dietary data collection and analysis.

Reporting for specific materials, systems and methods

We require information from authors about some types of materials, experimental systems and methods used in many studies. Here, indicate whether each material, system or method listed is relevant to your study. If you are not sure if a list item applies to your research, read the appropriate section before selecting a response.

Materials & experimental systems

n/a	Involved in the study	
☒	☐	Antibodies
☒	☐	Eukaryotic cell lines
☒	☐	Palaeontology and archaeology
☒	☐	Animals and other organisms
☐	☒	Clinical data
☒	☐	Dual use research of concern
☒	☐	Plants

Methods

n/a	Involved in the study	
☒	☐	ChIP-seq
☒	☐	Flow cytometry
☒	☐	MRI-based neuroimaging

Clinical data

Policy information about clinical studies

All manuscripts should comply with the ICMJE guidelines for publication of clinical research and a completed CONSORT checklist must be included with all submissions.

Clinical trial registration: NCT03848299

Study protocol: https://journals.plos.org/digitalhealth/article?id=10.1371/journal.pdig.0000389

Data collection: Data was collected from 1,014 participants across Switzerland between 2018-2022. Participants used the MyFoodRepo mobile app to record their daily food intake in real-time, under real-world conditions, without requiring clinical visits. A single stool sample was collected from each participant during their tracking phase, which was sequenced at Microsynth AG (Switzerland).

Outcomes:

Primary Outcome Measures:-

Blood glucose level: The participants will use the Flash Glucose Monitor FreeStyle Libre sensor and the FreeStyle Libre smartphone app from Abbott Diabetes Care to measure continuously during 14 days their glucose level.

Secondary Outcome Measures:-

Dietary intake: Food consumption information will be collected through the smartphone app myFoodRepo. The participants will track their dietary intake by taking pictures of all consumed food and beverages (including water).

Physical activity intensity: The intensity of the physical activity. By default, the participants will have to log manually this features in the study website. In case a participant prefers to use an activity tracker or a smartphone app he/she owns instead of manual logging, it will also be possible.

Physical activity frequency: The frequency of the physical activity. By default, the participants will have to log manually this features in the study website. In case a participant prefers to use an activity tracker or a smartphone app he/she owns instead of manual logging, it will also be possible.

Physical activity duration: The duration of the physical activity. By default, the participants will have to log manually this features in the study website. In case a participant prefers to use an activity tracker or a smartphone app he/she owns instead of manual logging, it will also be possible.

The sleep duration: The duration of sleep will be assessed. By default, the participants will have to log manually this features in the study website. In case a participant prefers to use an activity tracker or a smartphone app he/she owns instead of manual logging, it will also be possible.

The sleep timing: The timing of sleep will be assessed. By default, the participants will have to log manually this features in the study website. In case a participant prefers to use an activity tracker or a smartphone app he/she owns instead of manual logging, it will also be possible.

Microbiome composition: The participants will collect a stool sample. We will transfer the samples to Microsynth, a company specialized in DNA sequencing. They will extract the DNA from the stool sample and sequence the ribosomal DNA (16S rDNA) with Next-Generation Sequencing (NGS) technology. The composition of microbial communities in the gut of each participant will be determined by bioinformatic analysis.

Plants

Seed stocks: *Report on the source of all seed stocks or other plant material used. If applicable, state the seed stock centre and catalogue number. If plant specimens were collected from the field, describe the collection location, date and sampling procedures.*

Novel plant genotypes: *Describe the methods by which all novel plant genotypes were produced. This includes those generated by transgenic approaches, gene editing, chemical/radiation-based mutagenesis and hybridization. For transgenic lines, describe the transformation method, the number of independent lines analyzed and the generation upon which experiments were performed. For gene-edited lines, describe the editor used, the endogenous sequence targeted for editing, the targeting guide RNA sequence (if applicable) and how the editor was applied.*

Authentication: *Describe any authentication procedures for each seed stock used or novel genotype generated. Describe any experiments used to assess the effect of a mutation and, where applicable, how potential secondary effects (e.g. second site T-DNA insertions, mosiacism, off-target gene editing) were examined.*
